# Heterometallic Copper–Vanadium Compounds: Crystal Structures of Polymers [Cu(*im*)_4_(V_2_O_4_(*mand*)_2_)]_*n*_ and [Cu(*im*)_4_(V_2_O_4_((*S*)-*mand*)_2_)]_*n*_·2*n*H_2_O (*im* = imidazole, *mand* = mandelato^2−^)

**DOI:** 10.1007/s10870-019-00810-8

**Published:** 2019-10-17

**Authors:** Mária Šimuneková, Peter Schwendt, Róbert Gyepes, Lukáš Krivosudský

**Affiliations:** 1 Comenius University in Bratislava, Faculty of Natural Sciences, Department of Inorganic Chemistry, Mlynská dolina, Ilkovičova 6, 842 15 Bratislava, Slovakia; 2 Charles University, Faculty of Science, Department of Inorganic Chemistry, Hlavova 2030, 128 00 Prague, Czech Republic; 3 Universität Wien, Fakultät für Chemie, Institut für Biophysikalische Chemie, Althanstraße 14, 1090 Vienna, Austria

**Keywords:** Copper, Vanadate, Heterometallic complexes, IR spectroscopy, Crystal structure

## Abstract

**Abstract:**

Two new 1D polymeric heterometallic copper–vanadium compounds were prepared. The polymers are constructed from [Cu(*im*)_4_]^2+^ cations that are coordinated to two terminal oxido ligands of [V_2_O_4_(*mand*)_2_]^2−^ anions. The stronger coordination in [Cu(*im*)_4_V_2_O_4_(*mand*)_2_]_*n*_ (**1**) that contains the racemic mandelato ligand is manifested by a shorter Cu‒O bond distance 2.4095(12) Å, while the weaker interaction in [Cu(*im*)_4_(V_2_O_4_((*S*)-*mand*)_2_)]_*n*_·2*n*H_2_O (**2**) is exhibited by Cu‒O bond distances 2.4547(16) Å and 2.5413(16) Å. The vanadate anion in compound **2** carries only the (*S*)-enantiomer of the initial mandelic acid and differs from the anion in **1** in parallel *cis* orientation of the phenyl groups of the mandelato ligand. FT-IR spectroscopy was used for the confirmation of the coordination mode of mandelato ligand. Strong bands corresponding to the vibrations of carboxyl groups can be observed around 1650 and at 1344 cm^−1^. The stretching vibration of deprotonated hydroxyl group in the mandelato ligand occurs at 1045 and 1065 cm^−1^ for **1** and **2**, respectively. In addition, the very strong, characteristic band corresponding to *ν*(V=O) vibration can be observed at 931 cm^−1^ for **1** and 925 cm^−1^ for **2**, as well as in Raman spectrum.

**Graphic Abstract:**

The polymeric structures of two new vanadium-copper heterometallic complexes are constructed from [Cu(*imidazole*)_4_]^2+^ cations that are coordinated to two terminal oxido ligands of [V_2_O_4_(*mandelato*)_2_]^2−^ anions with different orientation of the phenyl groups depending on the chirality of the mandelato ligand. 
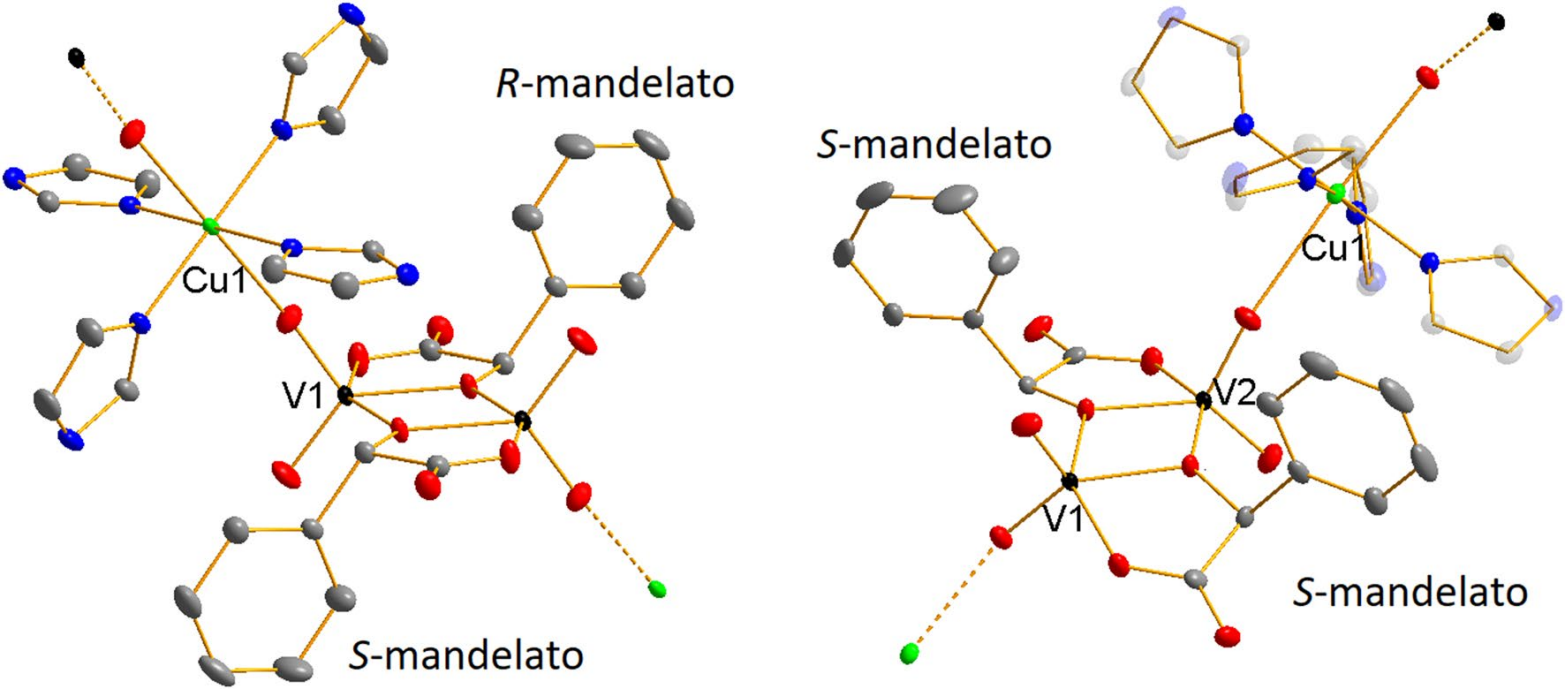

**Electronic supplementary material:**

The online version of this article (10.1007/s10870-019-00810-8) contains supplementary material, which is available to authorized users.

## Introduction

There are more than 6900 structures containing copper and imidazole in the Cambridge Structural Database (CCDC, April 2019) [1], and among those, 36 structures contain simultaneously vanadium. Omitting vanadium(IV) and mixed valence vanadium(IV)–vanadium(V) compounds, seventeen compounds remain that incorporate only vanadium(V). Most of these are composed of the oxovanadate ion V_4_O_12_^4−^ or (VO_3_)_*n*_^*n*−^ chain and a copper complex with substituted imidazoles as ligands [1]. Only three compounds contain unsubstituted imidazole (*im* = imidazole), namely enantiomers {[Λ–Cu(*en*)(*im*)_2_][VO_3_]_2_}_*n*_ and {[Δ–Cu(*en*)(*im*)_2_][VO_3_]_2_}_*n*_ [2] and [Cu(*im*)_4_]_2_(V_4_O_12_) [3]. There are only two compounds where an organic ligand is bound both to vanadium and copper atom: [{VO(O_2_)_2_(*im*)}_2_{*µ*–Cu(*im*)_4_}] [4] and {Cu(*im*)_4_[(VO_2_F_2_(*py*)]_2_} (*py* = pyridine) [5]. Both compounds described in this paper belong to this last group of rare compounds. [Cu(*im*)_4_(V_2_O_4_(*mand*)_2_]_*n*_ (**1**) and [Cu(*im*)_4_(V_2_O_4_((*S*)-*mand*)_2_)]_*n*_·2*n*H_2_O (**2**) (*mand* = mandelato(2–) ligand) were prepared as a part of our continual research on heterometallic transition metal–vanadium compounds with potential applications in asymmetric catalysis or development of new anode materials for batteries [4, 6–9]. Herein, we discuss the unexpected polymeric structures of **1** and **2** that differ significantly from the heretofore characterized, mostly chiral, compounds.

## Experimental

### Synthesis and Characterization

#### Materials and Methods

Chemicals and solvents were obtained from commercial sources: H_2_O_2_ (35%, p. a., Centralchem), CuCl_2_·2H_2_O (p. a., Lachema), KBr (for IR spectra, Lachema), imidazole (p. a., Lachema), *rac*-mandelic acid (for synth., Merck), (*S*)-mandelic acid (99% +, Acros Organics), dimethyl sulfoxide (DMSO, p. a., Penta), acetonitrile (99.5%, Centralchem). NH_4_VO_3_ (purum, Lachema) was purified according to [9].

Elemental analyses C,H,N were determined on a Vario MIKRO cube (Elementar). Vanadium was determined using ICP-MS (Perkin-Elmer Sciex Elan 6000) and copper was determined using F-AAS (Perkin Elmer 1100). Infrared spectra in KBr discs or spectra using the ATR technique were recorded on a Nicolet FTIR 6700 spectrometer. The Raman spectrum was recorded on the same instrument equipped with Nicolet NXR FT-Raman module (λ = 976 nm) and InGaAs detector.

#### Synthesis of [Cu(*im*)_4_(V_2_O_4_(*mand*)_2_)] (**1**)

NH_4_VO_3_ (0.233 g, 2 mmol) was dissolved in water (15 cm^3^) and H_2_O_2_ (35%, 0.2 cm^3^) and *rac*-H_2_*mand* (0.305 g, 2 mmol) was added. To the red solution obtained, the solution of CuCl_2_·2H_2_O (0.171 g, 1 mmol) and imidazole (0.409 g, 6 mmol) in acetonitrile (20 cm^3^) and water (10 cm^3^) was added under continuous stirring. The yellow precipitate was filtered off and the resulting dark filtrate was allowed to crystallize at 5 °C. Small green crystals were isolated after 48 h. Compound **1** is partially soluble in DMSO and insoluble in water, ethanol, acetonitrile.

*Anal*. Calc. for CuV_2_O_10_C_28_N_8_H_28_ (802.00 g/mol): C 41.93; H 3.52; N 13.97; V 12.70; Cu 7.92%; Found: C 41.92; H 3.42; N 14.05; V 12.18; Cu 7.92%.

#### Synthesis of [Cu(*im*)_4_(V_2_O_4_((*S*)-*mand*)_2_)]·2H_2_O (**2**)

NH_4_VO_3_ (0.233 g, 2 mmol) was dissolved in water (15 cm^3^) and H_2_O_2_ (35%, 0.2 cm^3^) and (*S*)-H_2_*mand* (0.305 g, 2 mmol) was added. To the red solution obtained, the solution of CuCl_2_·2H_2_O (0.171 g, 1 mmol) and imidazole (0.409 g, 6 mmol) in acetonitrile (20 cm^3^) and water (10 cm^3^) was added under continuous stirring. The yellow precipitate was filtered off and the resulting dark solution was allowed to crystallize at 5 °C. Dark violet crystals were isolated after several days. Compound **2** is soluble in DMSO and insoluble in water, ethanol, acetonitrile.

*Anal*. Calc. for CuV_2_O_12_C_28_N_8_H_32_ (838.03 g/mol): C 40.13; H 3.85; N 13.37; V 12.16; Cu 7.58%; Found: C 40.30%; H 3.55%; N 13.15%; V 11.66%; Cu 7.62%.

#### Structure Determination Procedures

Single-crystal X-ray diffraction data were collected using a Bruker VENTURE diffractometer and MoK_α_ primary radiation (λ = 0.71073 nm) at 120 K. Absorption correction was applied using SADABS [10]. The phase problem was solved with intrinsic phasing using SHELXT [11] and structure models were refined with SHELXL 2018 [12]. All non-hydrogen atoms were refined anisotropically, while all hydrogen atoms isotropically. Hydrogen atoms on carbon atoms were placed at idealized positions and hydrogen atoms on nitrogen atoms were refined with no restraints. The final structure models have been deposited with the Cambridge Crystallographic Data Centre (CCCDC) under deposition numbers 1922300 for **1** and 1922301 for **2**. These data can be obtained free of charge under https://www.ccdc.cam.ac.uk/structures/.

## Results and Discussion

### Synthesis

Crystals of **1** and **2** were obtained by crystallization from the NH_4_VO_3_–*rac*-mandelic acid/*S*-mandelic acid–CuCl_2_·H_2_O–imidazole–H_2_O_2_–H_2_O–CH_3_CN reaction solutions. Hydrogen peroxide prevented the reduction of vanadium(V) by mandelic acid, but it did not enter the final products. The bicomponent solvent H_2_O–CH_3_CN allowed the reaction of the initial reactants in a solution; and subsequently, acetonitrile acted as a precipitant enabling crystallization of the products.

### Crystal Structure

**Fig. 1:**
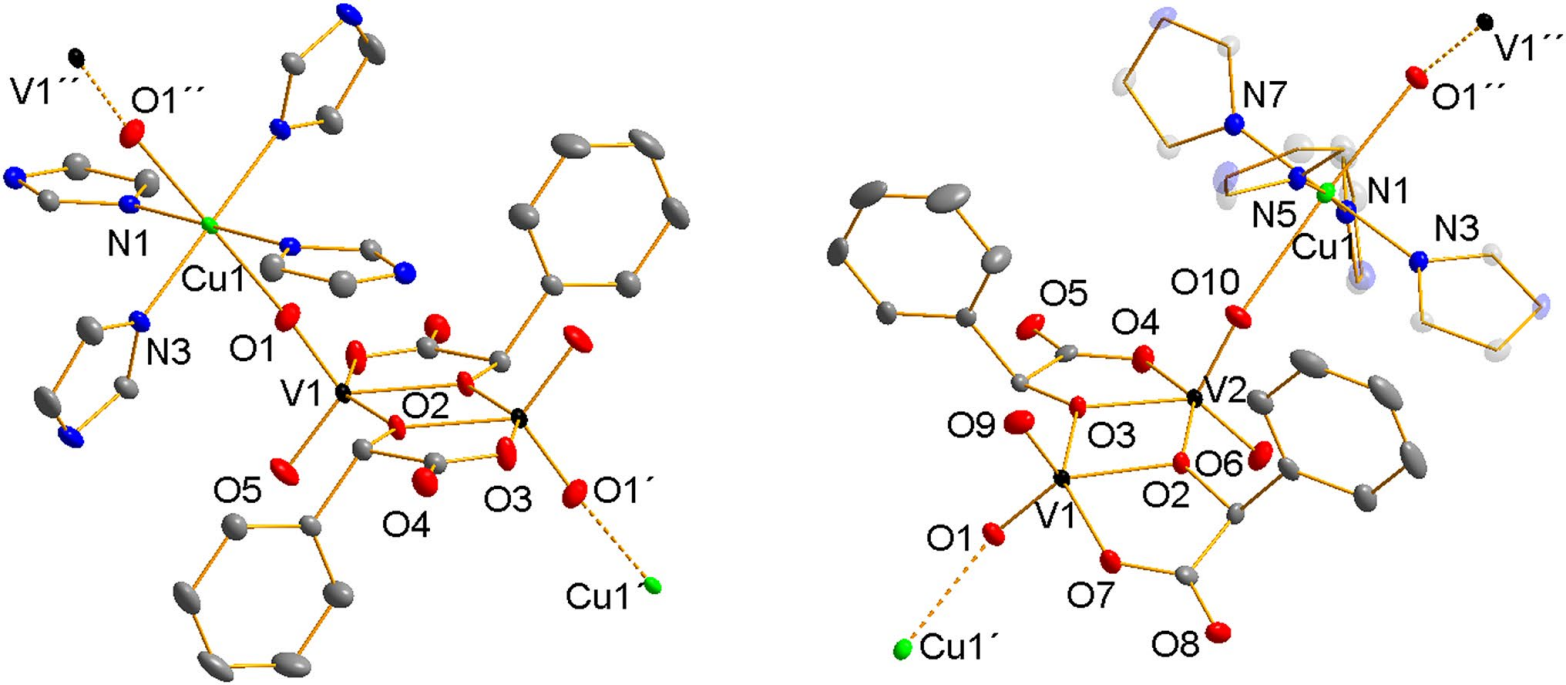
Molecular structures of the cationic {‒Cu(*im*)_4_‒}^2+^ and anionic {‒V_2_O_4_(*mand*)_2_‒}^2−^ components of the polymers found in **1** (left) and **2** (right). Displacement ellipsoids are shown at 50% probability level. Colors: V black, Cu green, O red, N blue, C gray. Hydrogen atoms and water molecules are omitted for clarity (Color figure online)

Table 1 summarizes crystal structure data and refinement details for compounds **1** and **2**. While compound **1** contains both enantiomers of the mandelic acid and thus crystallizes in the centrosymmetric crystal system *P*−1, compound **2** crystallizes in the non-enantiogenic Sohncke group *P*2_1_ because its polymeric chain is constructed from the (*S*)-*mand* ligand only. The two compounds do not differ in the coordination fashion of the central atoms of vanadium(V) and copper(II) (Fig. 1). In both cases, each vanadium atom is coordinated by two terminal oxido ligands, one oxygen atom coming from the carboxylate anion and two oxygen atoms that originate in the hydroxyl group of the mandelic acid. These oxygen atoms act as bridging ligands between two vanadium atoms of the {V_2_O_4_(*mand*)_2_}^2−^ fragment. The copper(II) central atom is coordinated by four nitrogen atoms of the imidazole ligands in the tetragonal plane. The apical positions are occupied by oxido ligands of the {V_2_O_4_((*S)*-*mand*)_2_}^2−^ fragment forming an infinite polymeric 1D chain. The oxido ligands coordinated to the Cu(II) centers are always in the *trans* position. Table 2 summarizes bond lengths and angles found in **1** and **2**. The Cu1‒O1 bond length in **1** is 2.4095(12) Å. In compound **2** there are two different weaker Cu‒O bonds: Cu1‒O10 2.455 Å and Cu1‒O1 2.541 Å. Slightly different coordination of the {V_2_O_4_(*mand*)_2_}^2−^ fragments to Cu(II) central atoms in 1 and 2 manifests itself also in different colors of the two compounds (violet vs. green).

**Table 1:** Crystal structure data and refinement details for compounds **1** and **2**

	**1**	**2**
CCDC code	1922300	1922301
Chemical formula	C_28_H_28_N_2_O_10_CuV_2_	C_28_H_32_N_8_O_12_CuV_2_
Formula weight	802.00	838.03
Temperature	120 K	120 K
Wavelength	0.71073 Å	0.71073 Å
Crystal system, space group	Triclinic, *P *− 1	Monoclinic, *P*2_1_
Unit cell dimensions	*a *= 9.3321(7) Å *α* = 117.008(2)° *b* = 9.6328(6) Å *β* = 103.821(2)° *c* = 10.4769(7) Å *γ* = 96.333(2)°	*a *= 9.9846(9) Å *b* = 17.3819(16) Å *β* = 103.821(2)° *c* = 10.4350(9) Å
Volume	788.32(9) Å^3^	1746.8(3) Å^3^
Z, Calculated density	1, 1.689 g cm^−3^	2, 1.593 g cm^−3^
Absorption coefficient	1.319 mm^−1^	1.199 mm^−1^
F(000)	407	854
Crystal size	0.23 × 0.21 × 0.16 mm	0.67 × 0.30 × 0.15 mm
Theta range for data collection	2.4–27.6°	2.3382–29.0370°
Limiting indices	−12 ≤*h * ≤12, −11 ≤*k * ≤12, −13 ≤*l * ≤13	−12 <=*h * ≤12, −22 ≤*k * ≤22, −13 <=*l * ≤13
Reflections collected/unique	21715/3614 [*R*_int_ = 0.027]	33506/7899 [*R*_int_ = 0.043]
Absorption correction	numerical from formula	numerical from formula
Max. and min. transmission	0.82 and 0.70	0.84 and 0.57
Refinement method	Full-matrix least-squares on *F*^2^	Full-matrix least-squares on *F*^2^
Data/restraints/parameters	3614/0/231	7899/6/489
Goodness-of-fit on *F*^2^	1.039	1.069
Final R indices [*I *> 2*σ*(*I*)]	*R*_1_ = 0.0259, *wR*_2_ = 0.0660	*R*_1_ = 0.0214, *wR*_2_ = 0.0525
R indices (all data)	*R*_1_ = 0.0287, *wR*_2_ = 0.0673	*R*_1_ = 0.0225, *wR*_2_ = 0.0531
Flack parameter	N/A	0.037(8)
Largest diff. peak and hole	0.45 and −0.33 e Å^−3^	0.21 and −0.45 e Å^−3^

**Table 2:** Structural parameters for compounds **1** and **2**

Bond lengths in Å			
**1**		**2**	
V_1_–O_5_	1.6258 (12)	V_1_–O_9_	1.6223 (19)
V_1_–O_1_	1.6275 (12)	V_1_–O_1_	1.6324 (16)
V_1_–O_3_^i^	1.9645 (12)	V_1_–O_3_	1.9561 (15)
V_1_–O_2_	1.9707 (11)	V_1_–O_7_	1.9698 (17)
V_1_–O_2_^i^	2.0165 (11)	V_1_–O_2_	2.0368 (16)
O_1_–Cu1	2.4095 (12)	V_2_–O_10_	1.6140 (17)
Cu_1_–N_1_^ii^	2.0050 (13)	V_2_–O_6_	1.6301 (18)
Cu_1_–N_1_	2.0050 (13)	V_2_–O_2_	1.9717 (16)
Cu_1_–N_3_^ii^	2.0267 (13)	V_2_–O_4_	1.9790 (17)
Cu_1_–N_3_	2.0267 (13)	V_2_–O_3_	2.0069 (16)
		Cu_1_–N_3_	1.989 (2)
		Cu_1_–N_7_	1.990 (2)
		Cu_1_–N_5_	1.9974 (19)
		Cu_1_–N_1_	2.0124 (19)

Bond angles in °			
**1**		**2**	
O_5_–V_1_–O_1_	108.89 (7)	O_9_–V_1_–O_1_	108.45 (10)
O_5_–V_1_–O_3_^i^	98.29 (6)	O_9_–V_1_–O_3_	104.09 (8)
O_1_–V_1_–O_3_^i^	98.85 (6)	O_1_–V_1_–O_3_	96.10 (7)
O_5_–V_1_–O_2_	100.10 (6)	O_9_–V_1_–O_7_	101.39 (9)
O_1_–V_1_–O_2_	100.31 (6)	O_1_–V_1_–O_7_	97.66 (8)
O3^i^–V_1_–O_2_	147.42 (5)	O_3_–V_1_–O_7_	145.37 (7)
O_5_–V_1_–O_2_^i^	128.91 (6)	O_9_–V_1_–O_2_	117.78 (8)
O_1_–V_1_–O_2_^i^	122.17 (6)	O_1_–V_1_–O_2_	133.66 (8)
O3^i^–V_1_–O_2_^i^	76.63 (5)	O_3_–V_1_–O_2_	70.55 (6)
O_2_–V_1_–O_2_^i^	70.87 (5)	O_7_–V_1_–O_2_	76.98 (7)
V_1_–O_1_–Cu_1_	163.94 (7)	O_10_–V_2_–O_6_	108.87 (10)
N_1_^ii^–Cu_1_–N_1_	180.0	O_10_–V_2_–O_2_	105.00 (8)
N_1_^ii^–Cu_1_–N_3_^ii^	92.01 (5)	O_6_–V_2_–O_2_	97.33 (9)
N_1_–Cu_1_–N_3_^ii^	87.99 (5)	O_10_–V_2_–O_4_	100.17 (8)
N_1_^ii^–Cu_1_–N_3_	87.99 (5)	O_6_–V_2_–O_4_	96.56 (8)
N_1_–Cu_1_–N_3_	92.01 (5)	O_2_–V_2_–O_4_	145.39 (7)
N_3_^ii^–Cu_1_–N_3_	180.00 (8)	O_10_–V_2_–O_3_	116.04 (8)
N_1_^ii^–Cu_1_–O_1_^ii^	88.45 (5)	O_6_–V_2_–O_3_	135.09 (8)
N_1_–Cu_1_–O_1_^ii^	91.55 (5)	O_2_–V_2_–O_3_	70.87 (6)
N_3_^ii^–Cu_1_–O_1_^ii^	88.61 (5)	O_4_–V_2_–O_3_	76.92 (7)
N_3_–Cu_1_–O_1_^ii^	91.39 (5)	N_3_–Cu_1_–N_7_	178.70 (8)
N_1_^ii^–Cu_1_–O_1_	91.55 (5)	N_3_–Cu_1_–N_5_	91.13 (8)
N_1_–Cu_1_–O_1_	88.45 (5)	N_7_–Cu_1_–N_5_	90.14 (8)
N_3_^ii^–Cu_1_–O_1_	91.39 (5)	N_3_–Cu_1_–N_1_	91.12 (8)
N_3_–Cu_1_–O_1_	88.61 (5)	N_7_–Cu_1_–N_1_	87.63 (8)
O_1_^ii^–Cu_1_–O_1_	180.0	N_5_–Cu_1_–N^1^	175.38 (8)

Symmetry codes for **1**: (i) − *x *+ 1, − *y *+ 1, − *z *+ 2; (ii) − *x *+ 1, − *y *+ 2, − *z *+ 2

In contradiction to the configuration of the coordinating terminal V=O groups, the phenyl residues of the mandelato ligands exhibit different orientation in each compound. In the centrosymmetric anion [V_2_O_4_(*rac*-*mand*)_2_]^2−^ in **1**, both enantiomers of the mandelic acid are present in the same dinuclear vanadium anion. As a result, the two individual ligands (*R*)- and (*S*)-*mand* are coupled by a center of symmetry and therefore the phenyl residues exhibit *trans* configuration. On the other hand, [V_2_O_4_((*S*)-*mand*)_2_]^2−^ in **2** does not lie on the center of symmetry and contains only one enantiomer of the mandelato ligand. This results in the configuration of the phenyl residues *cis*; a process that is also accompanied by overall decrease in symmetry of the central {V_2_C_4_O_10_} core of the anion.

**Fig. 2:**
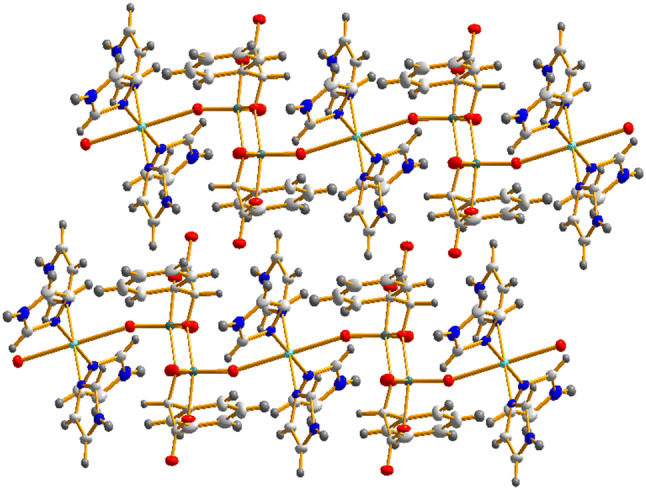
Propagation of the adjacent polymers along the crystallographic axis *c* in **1**

The adjacent polymeric chains in **1** interact through hydrogen bonds formed between the protonated nitrogen atoms of the imidazole rings and oxygen atoms O1 and O4 coming from the oxido and carboxylato ligands incorporated in the core of the [V_2_O_4_(*mand*)_2_]^2−^ anion. There are no significant π‒π interactions between the aromatic rings. Similar hydrogen bonding network can be found in **2**; in addition, in one position a water molecule is present interacting with two nitrogen atoms of the imidazole rings and two oxygen atoms O5 of the carboxylate ligand. The propagation of the adjacent polymers along the crystallographic axis *c* in **1** is displayed in Fig. 2.

### Spectroscopic Data Discussion

#### Infrared Spectra

**Fig. 3:**
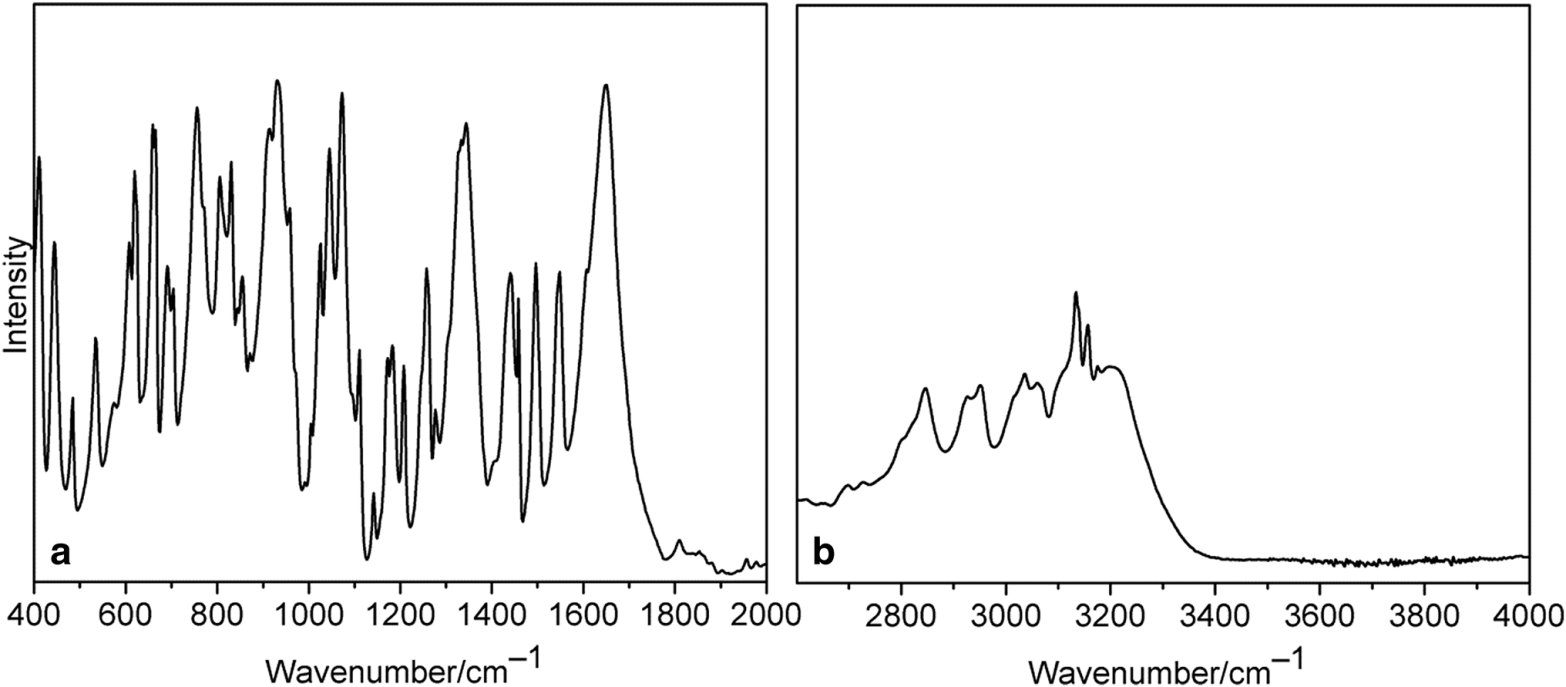
IR spectra of **1**: **a** in KBr disc, **b** ATR method

**Fig. 4:**
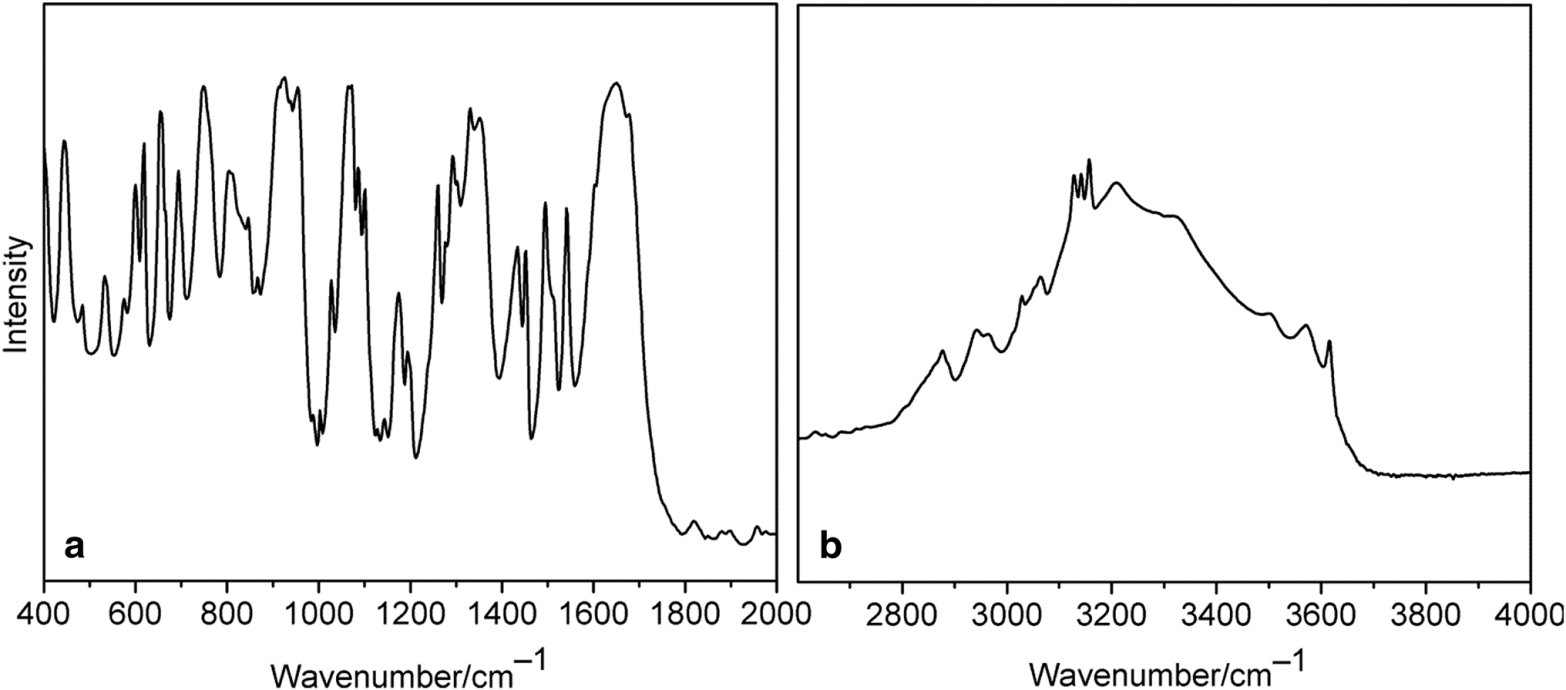
IR spectra of **2**: **a** in KBr disc, **b** ATR method

The FT-IR spectra of both **1** and **2** (Figs. 3, 4) exhibit a complicated pattern due to the presence of organic ligands. Nevertheless, some characteristic bands can be assigned. Thus, stretching vibrations *ν*(N–H) can be observed in the 3208–3316 cm^−1^ interval and *ν*(C–H) occurred between 3128 and 3175 cm^−1^ and 2846–3064 cm^−1^ for imidazole and mandelato ligand, respectively (Table 3). The O–H stretches of the water of crystallization for the compound **2** appear at 3615, 3571 and 3493 cm^−1^. The strong bands corresponding to the vibrations of carboxyl groups can be observed around 1650 and at 1344 cm^−1^, and the very strong band assignable to the coupled *ν*(CN), *ν*(CC) and *δ*(CCH) mode of imidazole [13] appears at 1072 cm^−1^. The stretching vibration of deprotonated hydroxyl group *ν*(C–O_h_) in the mandelato ligand occurs at 1045 and 1065 cm^−1^ for **1** and **2**, respectively. The very strong, characteristic band corresponding to *ν*(V=O) vibration can be observed at 931 cm^−1^ for **1** and 925 cm^−1^ for **2**. This band is of extreme intensity in the Raman spectrum of **1** (Fig. S1).

**Table 3:** Selected IR bands for **1** and **2**

Cu–*im*–*rac–mand* (**1**)	Cu–*im*–*S*-*mand* (**2**)	Assignment
	3615 m (ATR)	*ν*(OH) (H_2_O)
	3571 m(ATR)	
	3493 m (ATR)	
3209 s (KBr)	3316 m (ATR)	*ν*(NH) (imidazole)
	3208 s (ATR)	
3175 m	3157 s (ATR)	*ν*(CH) (imidazole)
3158 s	3142 s (KBr)	
3134 s	3128 s (KBr)	
3060 m	3064 m	*ν*(CH) (mandelato)
3035 m	3029 w	
2951 m	2942 m	
2927 w	2877 m	
2846 m		
1650 vs	1651 vs	*ν*(C=O_u_)
1344 s	1344 s	*ν*(C=O_c_)
1072 vs	1072 vs	*ν*(CN), *ν*(CC), *δ*(CCH) (imidazole)
1045 s	1065 vs	*ν*(C–O_h_)
931 vs	925 vs	*ν*(V=O)

*O*_*u*_ uncoordinated oxygen atom of carboxyl group, *O*_*c*_ coordinated oxygen atom of carboxyl group, *O*_*h*_ oxygen atom of hydroxyl group

## Electronic supplementary material

Below is the link to the electronic supplementary material.

Supplementary material 1 (DOCX 40 kb)
