# Supplementary material for: Heterometallic Copper–Vanadium Compounds: Crystal Structures of Polymers [Cu(im)4(V2O4(mand)2)]n and [Cu(im)4(V2O4((S)-mand)2)]n·2nH2O (im = imidazole, mand = mandelato2−)
Source: J Chem Crystallogr. 2019 Oct 17;50(4):373–80. doi: 10.1007/s10870-019-00810-8 (PMC7603452; doi:10.1007/s10870-019-00810-8)
Supplement: Supplementary file 1 — Supplementary material 1 (DOCX 40 kb) [file 10870_2019_810_MOESM1_ESM.docx]

**Supplementary data**

Heterometallic copper–vanadium compounds. Crystal structures of [Cu(*im*)_4_V_2_O_4_(*mand*)_2_)]*_n_* and [Cu(*im*)_4_V_2_O_4_(S-*mand*)_2_)]*_n_*∙2H_2_O (*im* = imidazole, *mand* = mandelato^2–^).

M. Šimuneková ^1^, P. Schwendt ^1^, R. Gyepes ^2^, L. Krivosudský ^1,3^

^1^ Comenius University in Bratislava, Faculty of Natural Sciences, Department of Inorganic Chemistry, Mlynská dolina, Ilkovičova 6, 842 15 Bratislava, Slovakia

^2^ Charles University, Faculty of Science, Department of Inorganic Chemistry, Hlavova 2030, 128 00 Praha, Czech Republic.

^3^ Universität Wien, Fakultät für Chemie, Institut für Biophysikalische Chemie, Althanstraße 14, Wien 1090, Austria

**

**

**Fig. S1** Raman spectrum of **1**
